# A Support Vector Machine based method to distinguish long non-coding RNAs from protein coding transcripts

**DOI:** 10.1186/s12864-017-4178-4

**Published:** 2017-10-18

**Authors:** Hugo W. Schneider, Taina Raiol, Marcelo M. Brigido, Maria Emilia M. T. Walter, Peter F. Stadler

**Affiliations:** 1 Department of Computer Science, University of Brasilia, ICC Central, Instituto de Ciências Exatas, Campus Universitario Darcy Ribeiro, Asa Norte, CEP: 70910-900, Brasilia, Brazil; 2 Gerência Regional de Brasilia (GEREB), Oswaldo Cruz Foundation (Fiocruz), Av. L3 Norte, Campus Universitário Darcy Ribeiro, Gleba A, Asa Norte, CEP: 70910-900, Brasília, Brazil; 3 Laboratory of Molecular Biology, University of Brasilia, Instituto de Ciencias Biologicas, Campus Universitario Darcy Ribeiro, Asa Norte, CEP: 70910-900, Brasilia, Brazil; 4 Bioinformatics Group, Department of Computer Science and Interdisciplinary Center for Bioinformatics, University of Leipzig, Hartelstrasse 16-18, Leipzig, D-04107 Germany

**Keywords:** Long non-coding RNA (lncRNA), lncRNA prediction with nucleotide pattern frequencies and ORF length, Support vector machine (SVM), Machine learning, Principal component analysis (PCA)

## Abstract

**Background:**

In recent years, a rapidly increasing number of RNA transcripts has been generated by thousands of sequencing projects around the world, creating enormous volumes of transcript data to be analyzed. An important problem to be addressed when analyzing this data is distinguishing between long non-coding RNAs (lncRNAs) and protein coding transcripts (PCTs). Thus, we present a Support Vector Machine (SVM) based method to distinguish lncRNAs from PCTs, using features based on frequencies of nucleotide patterns and ORF lengths, in transcripts.

**Methods:**

The proposed method is based on SVM and uses the first ORF relative length and frequencies of nucleotide patterns selected by PCA as features. FASTA files were used as input to calculate all possible features. These features were divided in two sets: (i) 336 frequencies of nucleotide patterns; and (ii) 4 features derived from ORFs. PCA were applied to the first set to identify 6 groups of frequencies that could most contribute to the distinction. Twenty-four experiments using the 6 groups from the first set and the features from the second set where built to create the best model to distinguish lncRNAs from PCTs.

**Results:**

This method was trained and tested with human (*Homo sapiens*), mouse (*Mus musculus*) and zebrafish (*Danio rerio*) data, achieving 98.21%, 98.03% and 96.09*%*, accuracy, respectively. Our method was compared to other tools available in the literature (CPAT, CPC, iSeeRNA, lncRNApred, lncRScan-SVM and FEELnc), and showed an improvement in accuracy by ≈3.00*%*. In addition, to validate our model, the mouse data was classified with the human model, and vice-versa, achieving ≈97.80*%* accuracy in both cases, showing that the model is not overfit. The SVM models were validated with data from rat (*Rattus norvegicus*), pig (*Sus scrofa*) and fruit fly (*Drosophila melanogaster*), and obtained more than 84.00*%* accuracy in all these organisms. Our results also showed that 81.2*%* of human pseudogenes and 91.7*%* of mouse pseudogenes were classified as non-coding. Moreover, our method was capable of re-annotating two uncharacterized sequences of Swiss-Prot database with high probability of being lncRNAs. Finally, in order to use the method to annotate transcripts derived from RNA-seq, previously identified lncRNAs of human, gorilla (*Gorilla gorilla*) and rhesus macaque (*Macaca mulatta*) were analyzed, having successfully classified 98.62%, 80.8% and 91.9*%*, respectively.

**Conclusions:**

The SVM method proposed in this work presents high performance to distinguish lncRNAs from PCTs, as shown in the results. To build the model, besides using features known in the literature regarding ORFs, we used PCA to identify features among nucleotide pattern frequencies that contribute the most in distinguishing lncRNAs from PCTs, in reference data sets. Interestingly, models created with two evolutionary distant species could distinguish lncRNAs of even more distant species.

**Electronic supplementary material:**

The online version of this article (doi:10.1186/s12864-017-4178-4) contains supplementary material, which is available to authorized users.

## Background

In recent years, thousands of sequencing projects around the world have been creating enormous volumes of RNA data, which has led to the discovery and description of a rapidly increasing number of non-coding RNAs (ncRNAs) in eukaryotic genomes [1–4]. NcRNAs are a highly heterogeneous group, ranging in length from about 20 bases in microRNAs and siRNAs [5] to “macroRNAs” spanning hundreds of kilobases [6, 7], known as long non-coding RNAs (lncRNAs). While the majority of ncRNAs seem to be spliced and processed similar to coding mRNAs, there is also a large body of unspliced transcripts [8, 9] and a vast number of small processing products [10]. The functions of ncRNAs are analogously diverse. In fact, they appear to be involved in virtually all the regulatory processes in the cell.

Although they are often pragmatically defined as transcripts of a more than 200 nucleotides in length, and without any apparent coding capacity, lncRNAs are still rather poorly understood [11–13]. Nevertheless, some classes, such as chromatin-associated long *intergenic* ncRNAs (lincRNAs) [14], as well as subgroups that are directly involved in transcriptional and post-transcriptional regulation [15–17], have been identified in high throughput analyses. An extensive literature links lncRNAs with a wide array of diseases [18–21], although the molecular mechanisms underlying lncRNA action are still largely unknown.

Distinguishing between protein coding transcripts (PCTs) and long non-coding transcripts (lncRNAs) is a surprisingly difficult task in practice, and there is still an ongoing controversy whether some or even the majority of the transcripts currently classified as “non-coding” can in fact be translated.

From a computational point of view, distinguishing PCTs from lncRNAs is a paradigmatic machine learning task, and several tools have become available for this purpose. Among these tools, CPC (Coding Potential Calculator) [22] and CPAT [23] have been developed to discriminate PCTs from ncRNAs. While CPC works well with known PCTs, it may tend to classify novel PCTs as ncRNAs, if they have not been recorded in protein databases [22]. The CPAT tool is based on logistic regression, and it uses four features based on ORFs.

Tools such as LncRNApred [24], lncRScan-SVM [25], DeepLNC [26] can predict lncRNAs. IseeRNA [27] was specially designed to predict lincRNAs. LncRScan-SVM and iSeeRNA are methods based on Support Vector Machines (SVM), trained with data from humans and mice, both presenting very good results. To predict lncRNAs, these two methods use GTF as input files, along with conservation data and some nucleotide patterns extracted from the sequences, to predict lncRNAs. LncRNApred is a method that was constructed using Random Forest, and features extracted from the sequence nucleotides to predict lncRNAs. DeepLNC was built using deep neural networks, and reported high accuracy to predict lncRNAs. Unfortunately, it is not clear which features were used, and the DeepLNC site presents an exception when any fasta file is submitted.

Recently, Wucher et al. [28] proposed FEELnc (FlExible Extraction of LncRNAs), a program to annotate lncRNAs based on a Random Forest model, trained with frequency nucleotide patterns and relaxed ORFs. They used FEELnc on a data set of canine RNA-seq samples, having improved the canine genome annotation with 10.374 novel lncRNAs.

Comprehensive reviews of these tools have been provided by Han et al. [25] and Guo et al. [29]. Similarly, Ventola et al. [30] studied features extracted from sequence data, those presented in the literature and some newly proposed features, in order to find signatures (groups of features) that can distinguish lncRNA transcripts from other classes, such as PCTs.

In general, the basic idea of the methods that use information of transcript nucleotides is to create a model to predict ncRNAs from known samples already stored in databases. Despite working well with the species for which they have been trained, these methods do not usually generalize for other organisms. In other words, these approaches are not capable of reliably predicting lncRNAs in a variety of species.

In addition, there are various databases containing lncRNAs (see Guo et al. [29] and Fritah et al. [31] for detailed reviews). Among them, Ensembl [32], NONCODE v. 4.0 [33], lncRNAdb [34], PLncDB [35], NRED [36] provide information on general and specific lncRNAs, while DIANA-LncBase [37] and lncRNADisease [38] present interactions among lncRNAs and other ncRNAs or proteins.

Moreover, in recent years, experimental and computational models have been developed to predict secondary and tertiary structures of lncRNAs, as explored in Yan et al. [39]. While the prediction of the lncRNAs’ secondary structures *in-vitro* has high-experimental costs, *in-silico* methods are low cost, but they exhibit high false-positive rates [29].

Although lncRNAs have very heterogenous characteristics [11–13], the previous described methods indicate that there are sets of features that allow researchers to distinguish lncRNAs from PCTs.

In this study, we present a SVM based method to distinguish lncRNAs from PCTs, using features extracted from transcript sequences: frequencies of nucleotide patterns selected by Principal Component Analysis (PCA) [40]; open reading frame (ORF) length; and ORF relative length. In addition, in order to analyze the performance of our method, we developed case studies with human, mouse and zebrafish data. We also compared results of our method to other tools found in the literature. To validate our model, we applied it to three different species (human, gorilla and rhesus macaque), as well as to human and mouse pseudogenes. Finally, we re-annotated data from Swiss-Prot, and annotated transcripts derived from RNA-seq data, reported in Necsulea et al. [41].

## Methods

### Data

Four data sets for training the models were obtained from Ensembl [32]: human (*Homo sapiens*) assemblies GRCh37 patch 13 (hg19, GENCODE 19) and GRCh38 patch 10 (hg38, GENCODE 26), mouse (*Mus musculus*) assembly GRCm38 patch 5 (mm10, GENCODE M13), zebrafish (*Danio rerio*) assembly GRCz10. These transcript FASTA files contain PCT and lncRNA sequences, while the classification was extracted from the transcript biotype, provided by Ensembl.

### The SVM based method

**Fig. 1:**
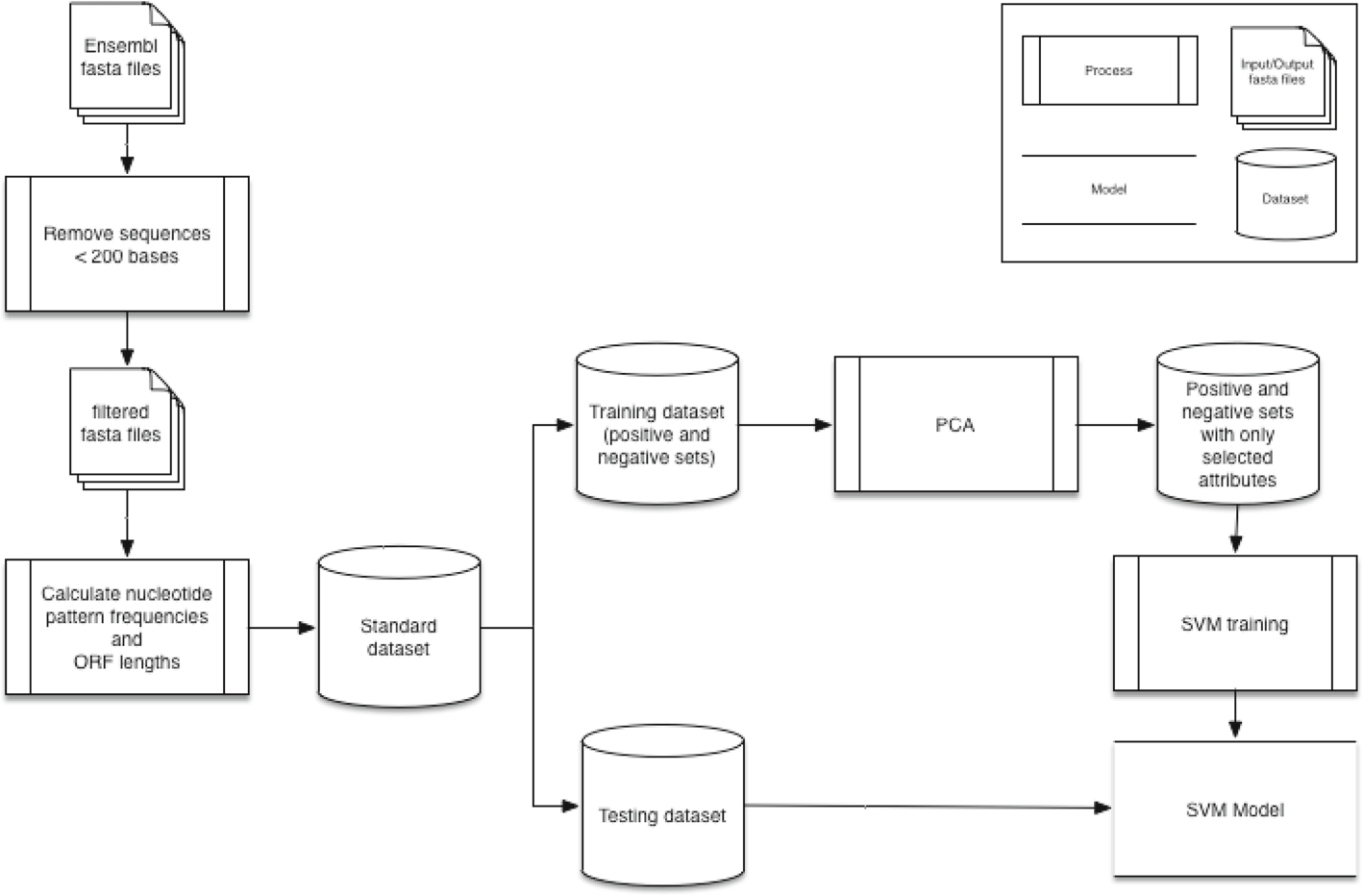
The method to distinguish lncRNAs from PCTs. The method is based on SVM and uses as attributes nucleotide pattern frequencies, chosen with the support of PCA, together with the first ORF relative length, a characteristic that informs the coding potential of a transcript

We propose a method based on SVM to distinguish lncRNAs from PCTs (see Fig. 1), using PCA to reduce the number of features calculated from the nucleotides of the transcripts.

First, a standard data set was created, removing all the sequences shorter than 200 bases from the original FASTA files. This standard data set contained, besides the transcripts (description and sequence), some calculated features (nucleotide pattern frequencies and ORF lengths) for each transcript, as follows. These features were divided in two sets: the first one contained the average frequency of the di-, tri- and tetra-nucleotide patterns in all the possible frames; and the second set contained the length and relative length of the first and the longest predicted ORFs. The relative length of an ORF is defined by its length divided by its corresponding transcript length.

The standard data set generated two other sets - training and testing, each composed of a positive set (containing lncRNAs) and a negative set (containing PCTs), of equal sizes. The training and the testing data sets were randomly generated, 75% for training and 25% for testing.

#### First set of features built with PCA

In the standard data set, there was a total of 336 different frequencies of nucleotide patterns in the first set: 16 di-nucleotide pattern frequencies; 64 tri-nucleotide pattern frequencies; and, 256 tetra-nucleotide pattern frequencies. We reduced the number of these possible features, having identified their relative importance, with the PCA method [40]. Thus, PCA was applied to all the nucleotide pattern frequencies of the training data set, to find how many, and which ones, would effectively help to distinguish between lncRNAs and PCTs.

The orthogonal transformation produced by PCA was used to calculate the “contribution” of each nucleotide pattern frequency. This orthogonal transformation is an *n*×*n* matrix with eigenvectors in its columns and features in its rows, where *n* = 336 frequencies of nucleotide patterns. We removed the *m* least significant columns from this matrix, obtaining a new *n*×(*n*−*m*) matrix, and calculated the Euclidean norm of the new vectors, also called loadings, represented by its columns. These norms are the contributions of the frequencies after the dimension reduction. This allowed to select sets of nucleotide pattern frequencies in the training data set.

The PCA indicated that a set of 10 features could explain about ≈65.0*%* of data, while a set of 60 features could explain about ≈95.0*%* of data. From this information, we created 6 groups of nucleotide pattern frequencies with sizes 10, 20, 30, 40, 50 and 60. The frequencies of nucleotide patterns that most contributed to the orthogonal transformation were selected to create each group. Each of these groups formed the first potential sets of features. The PCA results can be seen in Additional file 1.

#### Second set of features regarding ORFs

In addition, four sets of features were constructed, in order to find the best set of features regarding ORFs: the first ORF length and its relative length; the first ORF relative length; the longest ORF length and its relative length; and the longest ORF relative length.

#### Implementation

To implement the SVM method [42], a libSVM package [43] was used.

In order to find the best set of features, we combined the 6 sets of features found with the PCA (10,20,30,40,50 and 60 frequencies of nucleotide patterns) and the 4 sets of features described above (the first and the longest ORF lengths and their relative lengths), thereby creating 24 experiments.

In these 24 experiments, the grid search tool^1^ with 10-fold cross validation was used in the training data set, to define which experiment performed best. In each experiment, the best *C* and *γ* parameters were selected. The grid search results can be seen in Additional file 2.

#### Case studies

Four case studies were performed to evaluate the SVM method. We validated all the models created with species different from those used in the training phase, according to the following data sets: rat (*Rattus norvegicus*) assembly Rnor6.0, pig (*Sus Scrofa*) assembly Sscrofa10.2, and fruitfly (*Drosophila melanogaster*) assembly BDGP6. We also applied the models to human and mouse pseudogenes. In addition to this, we re-annotated two sequences from Swiss-Prot database [44], and annotated contigs derived from RNA-seq transcripts of human, gorilla and rhesus macaque, reported in Necsulea et al. [41].

## Results and discussion

### Human

In the first case study, only human data from the assemblies GRCh37 (hg19) and the GRCh38 (hg38) were used for training and testing. Our databases included 104,763 PCTs and 24,513 lncRNAs from GRCh37, and 102,915 PCTs and 28,321 lncRNAs from GRCh38. We filtered all the sequences shorter than 200 bases, having obtained 94,830 and 92,716 PCTs from GRCh37 and GRCh38 assemblies, respectively, and 24,266 and 28,024 lncRNAs from GRCh37 and GRCh38 assemblies, respectively.

To train the models, 18,200 PCTs and 18,200 lncRNAs were used from GRCh37, and 21,018 PCTs and 21,018 lncRNAs from GRCh38. GRCh37 testing data set included 6,066 PCTs and 6,066 lncRNAs, while the GRCh38 testing data set contained 7,006 PCTs and 7,006 lncRNAs.

**Fig. 2:**
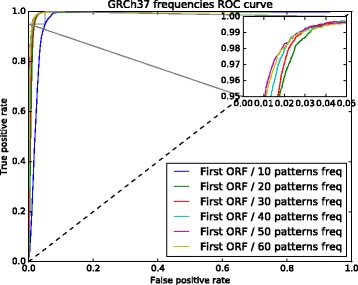
GRCh37 ROC curve used to select the set with the best nucleotide pattern frequencies. The model trained with a set composed of 50 nucleotide pattern frequencies performed slightly better than the other models

**Fig. 3:**
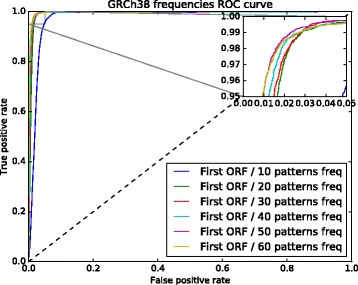
GRCh38 ROC curve used to select the set with the best nucleotide pattern frequencies. The model trained with a set composed of 50 nucleotide pattern frequencies performed slightly better than the other models

The 6 sets of nucleotide pattern frequencies selected with PCA were used to identify which one produced the best results. To do this, we used two ROC curves (see Figs. 2 and 3). These figures show the results of the models trained with the first ORF relative length and the 6 nucleotide pattern sets. The curve for the model trained with 50 nucleotide frequencies performed slightly better for both assemblies, GRCh37 and GRCh38.

The nucleotide pattern frequencies that achieved the best results for the human data are shown in Table 1. The nucleotide pattern frequencies for both GRCh37 and GRCh38 data sets were almost equal, the only difference being, “acg” and “gta”. We noted that both patterns are among the lowest PCA loadings, compared to all the other patterns.

**Table 1:** Selected nucleotide pattern frequencies for the human data

	GRCh37	GRCh38
1	aa, aaa, ac, aca, **acg**	aa, aaa, ac, aca, act
2	act, ag, aga, at, ata	ag, aga, at, ata, atc
3	atc, atg, att, ca, caa	atg, att, ca, caa, cac
4	cac, cag, cat, cc, cca	cag, cat, cc, cca, ccc
5	ccc, cg, cgc, ct, cta	cg, cgc, ct, cta, ctc
6	ctc, ctg, ga, gac, gag	ctg, ga, gac, gag, gc
7	gc, gcg, gg, ggg, gt	gcg, gg, ggg, gt, **gta**
8	gtc, gtg, ta, tac, tag	gtc, gtg, ta, tac, tag
9	tat, tc, tca, tct, tg	tat, tc, tca, tct, tg
10	tga, tgt, tt, ttg, ttt	tga, tgt, tt, ttg, ttt

GRCh37 and GRCh38 data sets were analyzed to identify 50 pattern frequencies with the highest PCA loadings. The patterns “acg” and “gta”, in bold, are the only difference. In the additional files, we listed these nucleotide pattern frequencies, ordered by PCA loadings

**Fig. 4:**
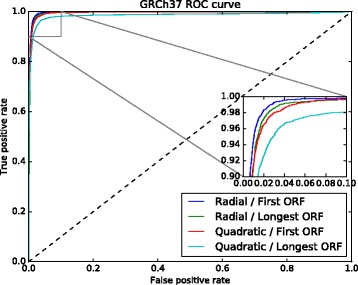
GRCh37 ROC curve used to select the best ORF relative length, and the kernel. The model trained with the first ORF relative length and the radial kernel obtained better results

**Fig. 5:**
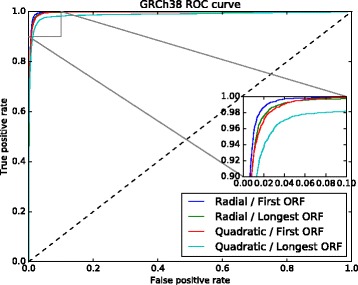
GRCh38 ROC curve used to select the best ORF relative lenght, and the kernel. The model trained with the first ORF relative length and the radial kernel obtained best results

Using these patterns, together with the first and longest ORF relative lengths as features, we trained 8 models with two kernels, radial and quadratic, having tested them with both data sets, GRCh37 and GRCh38. The results are shown in Table 2 and Figs. 4 and 5. The quadratic kernel achieved substantial accuracy in almost all the tests, while the radial kernel achieved very high accuracy in all of them.

**Table 2:** Results of the human case study

Test data set		
Model	GRCh37	GRCh38
Radial using GRCh37 and first ORF		
Sensitivity	**98.95%**	**99.43%**
Specificity	97.41*%*	97.23*%*
Accuracy	**98.18%**	98.33*%*
Radial using GRCh37 and longest ORF		
Sensitivity	98.09*%*	98.73*%*
Specificity	97.50*%*	97.55*%*
Accuracy	97.80*%*	98.14*%*
Quadratic using GRCh37 and first ORF		
Sensitivity	98.15*%*	98.83*%*
Specificity	96.60*%*	96.41*%*
Accuracy	97.38*%*	97.62*%*
Quadratic using GRCh37 and longest ORF		
Sensitivity	94.79*%*	95.54*%*
Specificity	97.23*%*	97.19*%*
Accuracy	96,01*%*	96.36*%*
Radial using GRCh38 and first ORF		
Sensitivity	89.86*%*	97.54*%*
Specificity	98.64*%*	**99.26%**
Accuracy	94.25*%*	**98.40%**
Radial using GRCh38 and longest ORF		
Sensitivity	98.37*%*	97.63*%*
Specificity	97.76*%*	97.58*%*
Accuracy	98.06*%*	97.61*%*
Quadratic using GRCh38 and first ORF		
Sensitivity	80.43*%*	98.66*%*
Specificity	**98.84%**	96.78*%*
Accuracy	89.63*%*	97.72*%*
Quadratic using GRCh38 and longest ORF		
Sensitivity	94.77*%*	95.08*%*
Specificity	97.66*%*	97.50*%*
Accuracy	96.21*%*	96.29*%*

We trained 8 models with two data sets, GRCh37 and GRCh38, to select the first, or the longest, ORF relative lengths (the length of the corresponding ORF divided by the length of the transcript). The better results for each data set are in bold

**Fig. 6:**
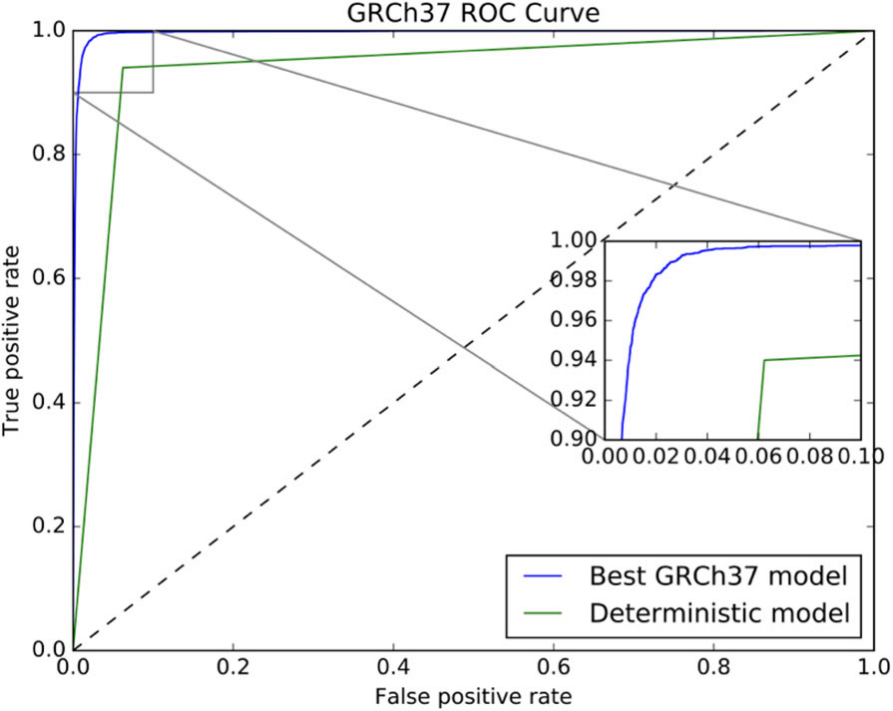
GRCh37 ROC curve showing the performance of the deterministic classifier compared to the best SVM model

In other results, the difference of the ORF relative length was very small when using the first and the longest ORF relative lengths. Although we were able to achieve very close values of accuracy, the first ORF relative length model presented higher sensitivity than the longest one. In addition, finding the first ORF (*O*(*n*)) has a lower time complexity when compared to finding the longest ORF (*O*(*n* ^2^)). From a biological point of view, the canonical model for translation initiation is the scanning model of the ribosome, which is finding the initial “atg” codon [45]. It is worth noting that, in our data sets, in ≈94*%* of the lncRNAs, the first ORF was different from the longest one, while in ≈93*%* of the PCTs, the first and the longest ORFs were the same. Using only this characteristic, we built a deterministic classifier to distinguish lncRNAs from PCTs, and compared it with the best SVM model (Fig. 6). This classifier achieved ≈93.5*%* accuracy. Thus, we decided to use the first ORF relative length as a feature in our models.

**Fig. 7:**
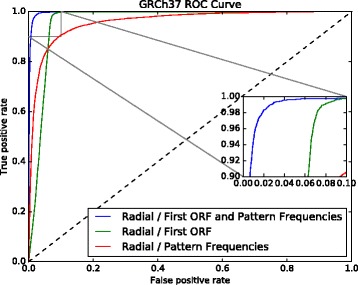
GRCh37 ROC curve showing the performance of a model trained with the first ORF relative length only, another model trained with the 50 selected nucleotide patterns frequencies, and a third model using all these features

**Fig. 8:**
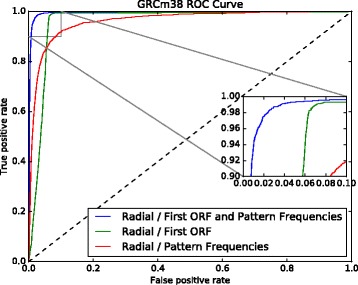
GRCh38 ROC curve showing the performance of a model trained with the first ORF relative length only, another model trained with the 50 selected nucleotide patterns frequencies, and a model using all these features

The contribution of each feature set was also investigated (Figs. 7 and 8). Each feature set can also distinguish lncRNAs from PCTs with high confidence. The model using only the first ORF achieved 92.90*%* accuracy in GRCh37 data set and 92.95*%* in GRCh38 data set, while the model using only the 50 frequencies of nucleotide pattern achieved an accuracy of 90.86% and 91.54*%*, respectively. These results confirm that ORF content is a key characteristic, as reported in the literature, and, also show that other features, such as sets of nucleotide pattern frequencies, can achieve similar performance in distinguishing lncRNAs from PCTs. However, we found that combining all the features in one model presented better results.

We compared our results with the methods and results presented by Sun et al. [27, 46], Han et al. [25], Pian et al. [24] and Wucher et al. [28], as shown in Table 3. Note that, in these comparisons, the same human assemblies were used. These results show that, in all the chosen metrics, our method presented better results.

**Table 3:** Results for models trained with human data

Test data set			
Method	GRCh37	GRCh38	NONCODE
Radial using GRCh37 and first ORF			
Sensitivity	**9** **8** **.** **9** **5** **%**	**9** **9** **.** **4** **3** **%**	***96.67%***
Specificity	97.41*%*	97.23*%*	-
Accuracy	**9** **8** **.** **1** **8** **%**	98.33*%*	-
Radial using GRCh38 and first ORF			
Sensitivity	89.86*%*	97.54*%*	88.75*%*
Specificity	**9** **8** **.** **6** **4** **%**	**9** **9** **.** **2** **6** **%**	-
Accuracy	94.25*%*	**9** **8** **.** **4** **0** **%**	-
CPC^a,e^			
Sensitivity	67.23*%*	69.90*%*	-
Specificity	97.62*%*	73.90*%*	-
Accuracy	82.43*%*	71.90*%*	-
CPAT^a,e^			
Sensitivity	94.60*%*	89.90*%*	-
Specificity	85.28*%*	92.40*%*	-
Accuracy	89.94*%*	91.20*%*	-
lncRScan-SVM^a^			
Sensitivity	93.88*%*	-	-
Specificity	89.20*%*	-	-
Accuracy	91.94*%*	-	-
iSeeRNA^b,c^			
Sensitivity	96.10*%*	-	-
Specificity	94.70*%*	-	-
Accuracy	95.40*%*	-	-
lncRNApred^d,f^			
Sensitivity	-	-	93.40*%*
Specificity	-	-	-
Accuracy	-	-	-
FEELnc^e^			
Sensitivity	-	92.30*%*	-
Specificity	-	91.50*%*	-
Accuracy	-	91.90*%*	-

Results in bold are the best for each test data set. Note that our method produced the best results

^a^Results obtained in Han et al. [25]

^b^Results obtained in Sun et al. [27]

^c^This method was created to classify only lincRNAs

^d^Results obtained in Sun et al. [24]

^e^Results obtained in Wucher et al. [28]

^f^We only considered *sensitivity*, since the negative test data was not clearly specified in the article

DeepLNC of Tripathi et al. [26] presented almost the same results, when compared to our method. In contrast to the other methods, we did not execute any experiment directly, since DeepLNC uses the lncipedia database [47], and does not clearly indicate the negative data set. We also attempted to use their method with our data set, but the web application (http://bioserver.iiita.ac.in/deeplnc/) presented an exception when submitting a fasta file, and failed to report any results. Notably, 98.21*%* of all the lncRNAs of the lncipedia database were correctly classified by our method.

Moreover, in order to verify the performance of our method in a highly curated set of lncRNAs and PCTs, we selected the best trained model to classify human data, the one trained with data from GRCh37, with 50 PCA selected nucleotide pattern frequencies and the first ORF relative length. This model was used to classify the highly curated data set of 5.322 lncRNAs reported by Nitsche et al. [48] and 5.322 PCTs randomly chosen from the Swiss-Prot reviewed database [44], but not including those annotated as *putative*, *hypothetical*, *unknown* and *predicted*. The model analyzed this data set with 96.15*%* accuracy, 99.72*%* sensitivity (5,307/5,322) and 92.58*%* specificity (4,927/5,322).

### Mouse

For the second case study, we used mouse transcript data, from the GRCm38 assembly, with 61,440 PCTs and 11,511 lncRNAs. Again, we removed all the sequences shorter than 200 nucleotides, which resulted in 57,191 PCTs and 11,347 lncRNAs. This data was randomly split in two data sets, a training data set with 8510 PCTs and 8510 lncRNAs, and a testing data set with 2837 PCTs and 2837 lncRNAs.

**Fig. 9:**
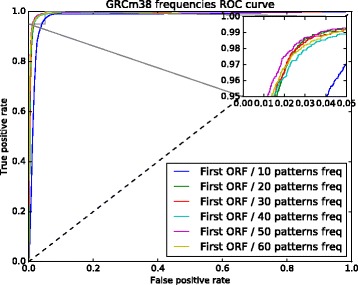
GRCm38 ROC curve used to select the best set of nucleotide pattern frequencies. The model trained with the set composed of 50 nucleotide pattern frequencies performed better than the other models

Models with the 6 nucleotide pattern sets together with the first ORF relative length were also used to find which set would perform better. The ROC curve in Fig. 9 shows that the model trained with 50 nucleotide frequencies performed better than the other models. The nucleotide pattern frequencies that achieved the best results for the mouse data are shown in Table 4.

**Table 4:** Selected nucleotide pattern frequencies for mouse data

	GRCm38
1	aa, aaa, ac, aca, acg
2	act, ag, aga, at, ata
3	atc, atg, att, ca, caa
4	cac, cag, cat, cc, cca
5	ccc, cg, cgc, ct, cta
6	ctc, ctg, ga, gac, gag
7	gc, gcg, gg, ggg, gt
8	gtc, gtg, ta, tac, tag
9	tat, tc, tca, tct, tg
10	tga, tgt, tt, ttg, ttt

GRCm38 data set was analyzed to identify the 50 pattern frequencies with the higher PCA loadings

**Fig. 10:**
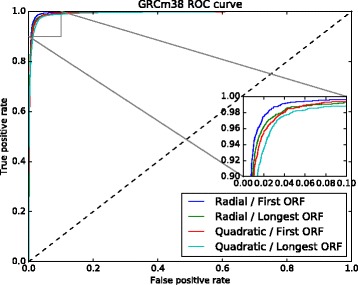
GRCm38 ROC curve used to select the best ORF relative lenght and the kernel. The model trained with the first ORF relative length and the radial kernel obtained the better results

Similar to the human case, using these nucleotide pattern frequencies, we also analyzed models trained with radial and quadratic kernels, using the first and the longest ORFs, as well as absolute and relative lengths. Analyzing the results, shown in Table 5 and in Fig. 10, we found that the best model was trained using the radial kernel, with features of the set of 50 frequencies of nucleotide patterns and the first ORF relative length.

**Table 5:** Results for models trained with mouse data

Test data set	
Model	GRCm38
Radial using GRCm38 and first ORF	
Sensitivity	**9** **8** **.** **7** **0** **%**
Specificity	96.96*%*
Accuracy	**9** **7** **.** **8** **3** **%**
Radial using GRCm38 and longest ORF	
Sensitivity	97.49*%*
Specificity	**9** **7** **.** **0** **3** **%**
Accuracy	97.26*%*
Quadratic using GRCm38 and first ORF	
Sensitivity	98.38*%*
Specificity	95.80*%*
Accuracy	97.09*%*
Quadratic using GRCm38 and longest ORF	
Sensitivity	96.51*%*
Specificity	96.99*%*
Accuracy	96.75*%*

Results in bold are the best ones for each test data set

**Fig. 11:**
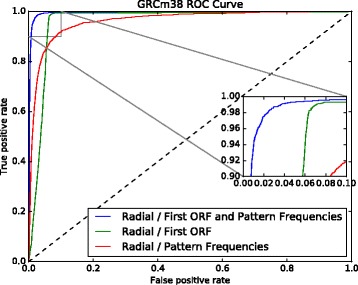
GRCm38 ROC curve showing the performance of a model trained with first ORF relative length only, another model trained with the 50 selected nucleotide patterns frequencies, and a third model using all these features

Again, the contribution of each feature category was investigated (Fig. 11). The model using only the first ORF achieved 93.52*%* accuracy, while the model using only the 50 frequencies of nucleotide patterns achieved an accuracy of 90.68*%*. Once more, ORF content is confirmed as a determinant characteristic, as well as a set of nucleotide pattern frequencies that achieved similar performance, to distinguish lncRNAs from PCTs. However, we found that combining all the features in one model improved performance.

The comparison of our results with those obtained by Sun et al. [27, 46], Han et al. [25], Pian et al. [24] and Wucher et al. [28] (see Table 6), shows that our method achieved better sensitivity and accuracy than the other methods, although the specificity was 1.41*%* lower than CPC, despite a 23.24*%* higher sensitivity in this case. Therefore, our method presented better performance in distinguishing lncRNAs from PCTs in mouse transcript data, when compared to the other tools.

**Table 6:** Results for models trained and tested with mouse data

Test data set	
Method	GRCm38 (mm10)
Radial using GRCm38 and first ORF	
Sensitivity	**9** **8** **.** **7** **0** **%**
Specificity	96.96*%*
Accuracy	**9** **7** **.** **8** **3** **%**
CPC^a^	
Sensitivity	75.46*%*
Specificity	**9** **8** **.** **3** **7** **%**
Accuracy	86.91*%*
CPAT^a^	
Sensitivity	95.34*%*
Specificity	88.17*%*
Accuracy	91.76*%*
lncRScan-SVM^a^	
Sensitivity	95.29*%*
Specificity	89.14*%*
Accuracy	92.21*%*
iSeeRNA^b,c^	
Sensitivity	94.20*%*
Specificity	92.70*%*
Accuracy	93.45*%*
FEELnc^d^	
Sensitivity	94.10*%*
Specificity	93.80*%*
Accuracy	93.90*%*

Results in bold are the best ones for each test data set

^a^Results obtained in Han et al. [25]

^b^Results obtained in Sun et al. [27]

^c^This method was created to classify only lincRNAs

^d^Results obtained in Wucher et al. [28]

### Human and Mouse

This case study was analyzed to verify if a cross species model would better distinguish lncRNAs from PCTs than the previously tested single species models.

In this case study, we used the same training and testing data from the previous case studies to build the training and testing data sets. We combined data from GRCh37 with GRCm38 and from GRCh38 with GRCm38.

First, we selected the 50 nucleotide pattern frequencies to build the models (see Table 7). The least significant patterns (lowest PCA loading), “cca” and “gac”, were the only differences in these sets.

**Table 7:** Selected nucleotide pattern frequencies for human and mouse data

	GRCh37 and GRCm38	GRCh38 and GRCm38
1	aa, aaa, ac, aca, acg	aa, aaa, ac, aca, acg
2	act, ag, aga, at, ata	act, ag, aga, at, ata
3	atc, atg, att, ca, caa	atc, atg, att, ca, caa
4	cac, cag, cat, cc, **cca**	cac, cag, cat, cc, ccc
5	ccc, cg, cgc, ct, cta	cg, cgc, ct, cta, ctc
6	ctc, ctg, ga, gag, gc	ctg, ga, **gac**, gag, gc
7	gcg, gg, ggg, gt, gtc	gcg, gg, ggg, gt, gtc
8	gtg, ta, tac, tag, tat	gtg, ta, tac, tag, tat
9	tc, tca, tcg, tct, tg	tc, tca, tcg, tct, tg
10	tga, tgt, tt, ttg, ttt	tga, tgt, tt, ttg, ttt

GRCh37, GRCh38 and GRCm38 data sets were analyzed to identify the 50 pattern frequencies with the highest PCA loadings. The patterns “cca” and “gac”, in bold, are the only differences

Using these patterns, we trained models with the first and the longest ORF relative lengths. The results are shown in Table 8.

**Table 8:** Results of the human and mouse case study

Test data set			
Model	GRCh37	GRCh38	GRCm38
Radial using GRCh37, GRCm38 and first ORF			
Sensitivity	**9** **8** **.** **8** **6** **%**	**9** **9** **.** **4** **2** **%**	98.51*%*
Specificity	97.56*%*	**9** **7** **.** **6** **9** **%**	97.54*%*
Accuracy	**9** **8** **.** **2** **1** **%**	**9** **8** **.** **5** **5** **%**	98.02*%*
Radial using GRCh37, GRCm38 and longest ORF			
Sensitivity	98.05*%*	98.67*%*	97.60*%*
Specificity	97.53*%*	97.59*%*	97.54*%*
Accuracy	97.79*%*	98.13*%*	97.57*%*
Radial using GRCh38, GRCm38 and first ORF			
Sensitivity	91.22*%*	99.24*%*	**9** **8** **.** **6** **6** **%**
Specificity	**9** **8** **,** **6** **5** **%**	97.46*%*	97.41*%*
Accuracy	94.93*%*	98.35*%*	**9** **8** **.** **0** **3** **%**
Radial using GRCh38, GRCm38 and longest ORF			
Sensitivity	98.31*%*	98.20*%*	98.23*%*
Specificity	97.83*%*	97.63*%*	**9** **7** **.** **7** **4** **%**
Accuracy	98.07*%*	97.91*%*	97.98*%*

We trained four models with two data sets, GRCh37/GRCm38 and GRCh38/GRCm38, and also compared the selection of two attributes, first and longest ORF relative lengths. The best results for each test data set, GRCh37, GRCh38 and GRCm38, are in bold

We noticed a small improvement in accuracy using the bi-species model, when compared to the single species model. These results suggest that a multi-species model can slightly improve distinguishing lncRNAs from PCTs, when compared to a single species model.

### Mouse and Zebrafish

The last case study was performed to evaluate our method when creating a multi-species model with data from two evolutionary distant species, together with a fewer number of annotated lncRNAs. To do this, we used mouse (GRCm38) and zebrafish (GRCz10).

The same training and testing data sets from the mouse case study were used, together with data from GRCz10, 2775 PCTs and 2775 lncRNAs for training, and 926 PCTs and 926 lncRNAs for testing.

The 50 nucleotide pattern frequencies selected by the PCA are shown in Table 9. These 50 patterns and the first ORF relative length were used to create the SVM model, which obtained the results presented in Table 10.

**Table 9:** Selected nucleotide pattern frequencies from mouse and zebrafish

	GRCm38 and GRCz10
1	aa, aaa, ac, aca, acg
2	act, ag, aga, at, ata
3	atc, atg, att, ca, caa
4	cac, cag, cat, cc, cca
5	ccc, cg, cgc, ct, cta
6	ctc, ctg, ga, gag, gc
7	gcg, gg, ggg, gt, gtc
8	gtg, ta, tac, tag, tat
9	tc, tca, tcg, tct, tg
10	tga, tgt, tt, ttg, ttt

GRCm38 and GRCz10 data sets were analyzed to identify the 50 pattern frequencies with the highest PCA loadings

**Table 10:** Results for the mouse and zebrafish case study. We trained one model with two data sets, GRCm38 and GRCz10

Test data set		
Model	GRCm38	GRCz10
Radial using GRCm38, GRCz10 and first ORF		
Sensitivity	98.56*%*	97.19*%*
Specificity	96.86*%*	95.00*%*
Accuracy	97.71*%*	96.09*%*

Once again, the results show that we can use the same method on different sets of species, creating a multi-species model to distinguish lncRNAs from PCTs, with high accuracy.

### Model validation

To validate our method, we used the best model of each case study to distinguish lncRNAs from PCTs in data sets of species that were not used in the SVM training. The objective was to analyze under- and overfitting, and also whether the models could distinguish lncRNAs from PCTs in data sets of evolutionarily close and distant species.

Besides the data sets used in each case study, we used data from pig (Sscrofa10.2) - 205 lncRNAs and 205 PCTs, rat (Rnor6.0) - 3537 lncRNAs and 3537 PCTs, and fruit fly (BDGP6) - 2776 lncRNAs and 2776 PCTs. All the results are shown in Table 11.

**Table 11:** Comparison of all the results for each species, together with their corresponding performances

Test data set							
Model	GRCh37	GRCh38	GRCm38	Rnor6.0	Sscrofa10.2	GRCz10	BDGP6
Radial using GRCh37 and first ORF							
Sensitivity	**9** **8** **.** **9** **5** **%**	**9** **9** **.** **4** **3** **%**	**9** **8** **.** **7** **2** **%**	94.16*%*	78.89*%*	95.19*%*	93.17*%*
Specificity	97.41*%*	97.23*%*	97.04*%*	94.90*%*	89.28*%*	95.23*%*	99.78*%*
Accuracy	98.18*%*	98.33*%*	97.88*%*	94.53*%*	84.08*%*	95.21*%*	96.47*%*
Radial using GRCh38 and first ORF							
Sensitivity	89.86*%*	97.54*%*	90.07*%*	78.13*%*	55.28*%*	74.68*%*	80.87*%*
Specificity	**9** **8** **.** **6** **4** **%**	**9** **9** **.** **2** **6** **%**	98.51*%*	**9** **7** **.** **8** **9** **%**	95.93*%*	98.45*%*	99.91*%*
Accuracy	94.25*%*	98.40*%*	94.29*%*	88.01*%*	75.60*%*	86.56*%*	88.67*%*
Radial using GRCm38 and first ORF							
Sensitivity	98.50*%*	98.90*%*	98.70*%*	93.85*%*	**7** **9** **.** **4** **0** **%**	95.14*%*	94.31*%*
Specificity	97.09*%*	96.93*%*	96.96*%*	94.91*%*	**8** **9** **.** **4** **3** **%**	94.70*%*	99.96*%*
Accuracy	97.79*%*	97.91*%*	97.83*%*	94.38*%*	**8** **4** **.** **4** **1** **%**	94.92*%*	96.97*%*
Radial using GRCh37, GRCm38 and first ORF							
Sensitivity	98.86*%*	99.42*%*	98.51*%*	93.11*%*	76.38*%*	94.62*%*	91.30*%*
Specificity	97.56*%*	97.69*%*	**9** **7** **.** **5** **4** **%**	95.39*%*	89.94*%*	95.63*%*	99.76*%*
Accuracy	**9** **8** **.** **2** **1** **%**	**9** **8** **.** **5** **5** **%**	98.02*%*	94.25*%*	83.16*%*	95.12*%*	95.53*%*
Radial using GRCh38, GRCm38 and first ORF							
Sensitivity	91.22*%*	99.24*%*	98.66*%*	81.00*%*	55.28*%*	77.17*%*	74.95*%*
Specificity	98.65*%*	97.46*%*	97.41*%*	97.81*%*	95.85*%*	**9** **8** **.** **7** **4** **%**	**9** **9** **.** **9** **2** **%**
Accuracy	94.93*%*	98.35*%*	**9** **8** **.** **0** **3** **%**	89.40*%*	75.56*%*	87.95*%*	87.43*%*
Radial using GRCm38, GRCz10 and first ORF							
Sensitivity	98.71*%*	99.10*%*	98.56*%*	**9** **4** **.** **6** **4** **%**	75.89*%*	**9** **7** **.** **1** **9** **%**	**9** **8** **.** **5** **7** **%**
Specificity	96.89*%*	96.72*%*	96.86*%*	94.69*%*	89.87*%*	95.00*%*	99.65*%*
Accuracy	97.80*%*	97.91*%*	97.71*%*	**9** **4** **.** **6** **7** **%**	82.88*%*	**9** **6** **.** **0** **9** **%**	**9** **9** **.** **1** **1** **%**

The best results for each species are in bold. In the columns are the test data set: human GRCh37 and GRCh38; mouse GRCm38; rat Rnor6.0; pig Sscrofa10.2; zebrafish GRCz10; and fruitfly BDGP6

From these results, we can see that none of the models are overfitted, since they were able to be applied to different species with high accuracy. The models that used GRCh38 data led to worse performance for evolutionarily distant species, especially when compared to models that used data from GRCh37. The newly 3808 annotated lncRNAs probably contribute to a model more fitted to evolutionarily close species.

The pig data set obtained the worst classification. These results could be explained by the small number of sequences in the data set, and also by the fact that this is not a model organism, so possibly this data is not curated enough. Nonetheless, our method can be used to improve the quality of lncRNA annotation in this species.

On the other hand, it is interesting to note that a multi-species model can improve the accuracy when compared to a single species model, as can be seen in Table 11. The accuracy was slightly improved when the GRCh37/GRCm38 model was used to distinguish lncRNAs from PCTs in the human GRCh37 data set. Interestingly, a model created with two evolutionary distant species - mouse and zebrafish - was able to distinguish lncRNAs of the fruit fly, which is an even more distant species.

Finally, we used human and mouse pseudogenes (in GTF files), having predicted 81.2*%* (12,033 from a total of 15,494) pseudogenes of the human genome, and 91.7*%* (6832 from a total of 7453) pseudogenes of the mouse genome. It is remarkable that there is such a large number of predicted pseudogenes as lncRNA, since pseudogenes are derived from ancient PCTs, and diverge slowly after their generation, losing coding capacity and potential regulatory signal [49]. Nevertheless, our method distinguishes pseudogenes from *bona fide* PCTs.

### PCTs re-annotation and RNA-seq annotation

The GRCh38 model was used to search for lncRNAs among *putative*, *hypothetical*, *unknown* and *predicted* human PCTs in the Swiss-Prot reviewed database [44]. We found 1245 sequences longer than 67 animo-acids (201 bases). To find the corresponding nucleotide sequences, we used the EMBL reference of each entry of the Swiss-Prot database. All these sequences were trimmed, in order to begin with a start codon, because we found sequences that were 5^′^ UTR long. This avoids introducing bias by the first ORF relative length in the discrimination between lncRNAs and PCTs. Our method found 231 candidates. From these, we focused in 21 candidates - those that had more than a 90% probability of being lncRNA, and shorter than 2000 bases. After analyzing the EMBL and Swiss-Prot databases and the sequences themselves, we found 2 putative PCTs with multiple “atg” at the 5’ UTR, and also with annotation warnings about *dubious prediction*. Thus, both sequences listed in Additional file 3, could be re-annotated as lncRNAs with high probability.

In addition, we also used transcripts derived from RNA-seq data to validate our model against annotated lncRNAs, as reported by Necsulea et al. [41]. They presented 11,890, 912 and 12,056 lncRNAs from human, gorilla (*Gorilla gorilla*) and rhesus macaque (*Macaca mulatta*), respectively. Our GRCh37 model correctly classified 11,726 (98.62*%*), 737 (80.81*%*) and 11,086 (91.95*%*) lncRNAs from human, gorilla and rhesus macaque, respectively.

## Conclusion

In this article, we presented an SVM based method to distinguish long non-coding RNAs (lncRNAs) from protein coding transcripts (PCTs), using features from the nucleotide patterns (frequencies of di-, tri- and tetra-nucleotides) of transcripts, chosen with the support of Principal Component Analysis (PCA), together with ORF length and ORF relative length.

We trained and tested our method with data of human, mouse and zebrafish, obtaining high performance. The best results were an accuracy of 98.18*%* with human transcripts, 97.83*%* with mouse transcripts and 96.09*%* with zebrafish transcripts. We compared our results with other methods in the literature (CPAT, CPC, iSeeRNA, lncRNApred, lncRScan-SVM and FEELnc) and found we had obtained better results.

To validate our model, we first classified the mouse data with the human model, and vice-versa, obtaining accuracy of ≈97.8*%* in both cases, showing that our model is not overfitted, and can be used with evolutionarily close species. We also validated the multi-species models human/mouse and mouse/zebrafish, which also produced excellent results. Next, we tested our models with data from rat, pig and fruit fly, having obtained accuracies from 84 to ≈99*%* in all these organisms. Our method classified 81.2*%* of human pseudogenes and 91.7*%* of mouse pseudogenes as non-coding, and also found 2 uncharacterized sequences, among 1245, in the Swiss-Prot reviewed database, indicating a high probability of being lncRNAs. Furthermore, the method successfully annotated the majority of the assembled transcripts derived from RNA-seq data from human (98,62*%*), gorilla (80,81*%*) and rhesus macaque (91,95*%*).

We intend to investigate if a semi-supervised learning method could reduce the size of the training data sets, while simultaneously maintaining high accuracy in the testing phase. This could be very useful to train models for organisms with a small amount of known lncRNA transcripts. Lastly, novel features (see Ventola et al. [30]) could be used in machine learning methods, also indicating potential biological characteristics of lncRNAs.

## Endnote

^1^ A Python script to find a model with *C* and *γ* parameters presenting the best accuracy, which is part of the libSVM package.

## Additional files

Additional file 1 This file shows the results of the PCA analysis. The sheets are organized by data sets and PCA results. For example, "GRCh37 PCA Explained" contains the explained data for each component obtained from the GRCh37 data. The PCA results are "PCA Explained", "Loadings" and "Attributes", containing the screeplot of the explained data, the loadings of the nucleotide pattern frequencies, and the selected attributes, respectively. This three sheets are available for each case study, GRCh37, GRCh38, GRCm38, GRCh37/GRCm38, GRCh38/GRCm38 and GRCm30/GRCz10. (XLSX 389 kb)

Additional file 2 Grid search results to find good *C* and *γ* parameters for the SVM method, for each case study. Each image in this file represents the best selection of parameters for each case study. The attributes used in this grid search were the set of 50 nucleotide pattern frequencies, the first and the longest ORF relative lengths. (PDF 130 kb)

Additional file 3 Sequences of the Swiss-Prot reviewed database not characterized, identified by our method with a high probability of being lncRNAs. (Fa 3.62 kb)

## Abbreviations

lncRNA: Long non-coding RNA
lincRNA: Long intergenic non-conding RNA
ncRNA: Non-coding RNA
ORF: Open reading frame
PCT: Protein coding transcript
PCA: Principal component analysis
ROC: Receiver operating characteristic
SVM: Support vector machine
