# Supplementary material for: A Support Vector Machine based method to distinguish long non-coding RNAs from protein coding transcripts
Source: BMC Genomics. 2017 Oct 18;18:804. doi: 10.1186/s12864-017-4178-4 (PMC5648457; doi:10.1186/s12864-017-4178-4)
Supplement: Supplementary file 2 — Grid search results to find good C and γ parameters for the SVM method, for each case study. Each image in this file represents the best selection of parameters for each case study. The attributes used in this grid search were the set of 50 nucleotide pattern frequencies, the first and the longest ORF relative lengths. (PDF 130 kb) [file 12864_2017_4178_MOESM2_ESM.pdf]

This document contains all results obtained by the libSVM grid search. This results were used to determine the best C and γ parameters for each model.

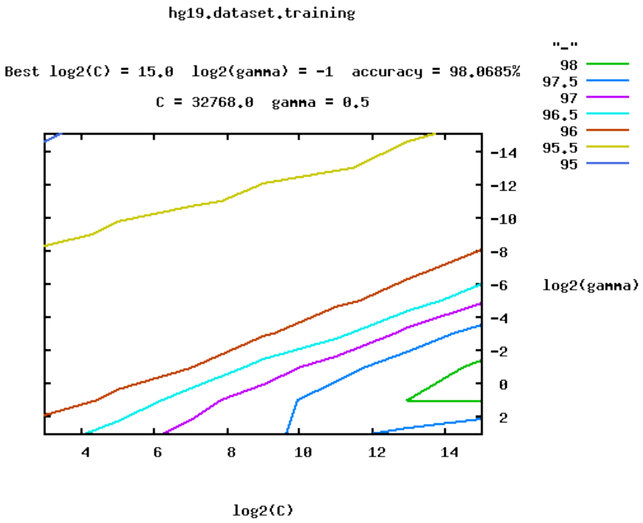

Figure 1: GRCh37 data using first ORF.

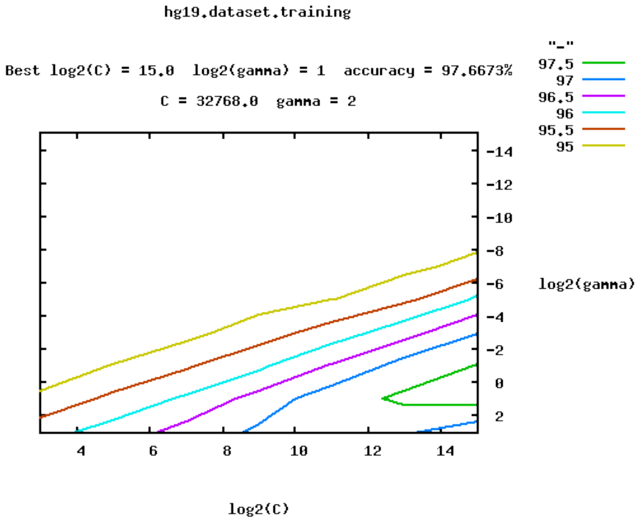

Figure 2: GRCh37 data using longest ORF.

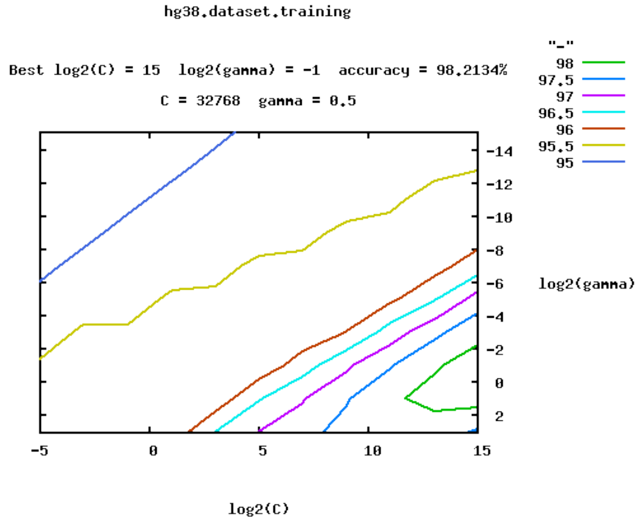

Figure 3: GRCh38 data using first ORF.

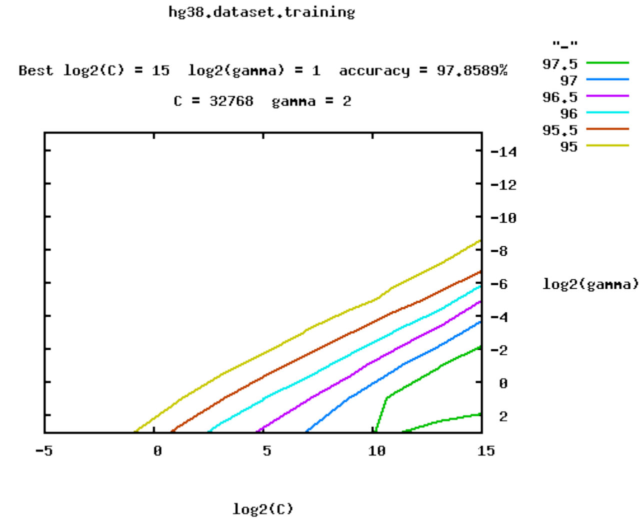

Figure 4: GRCh38 data using longest ORF.

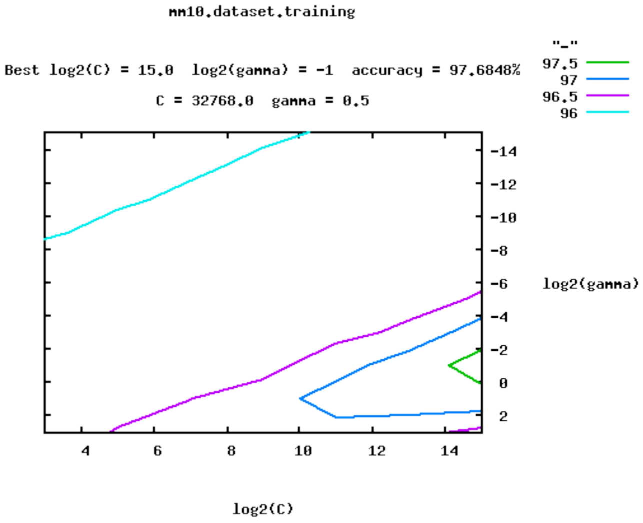

Figure 5: GRCm38 data using first ORF.

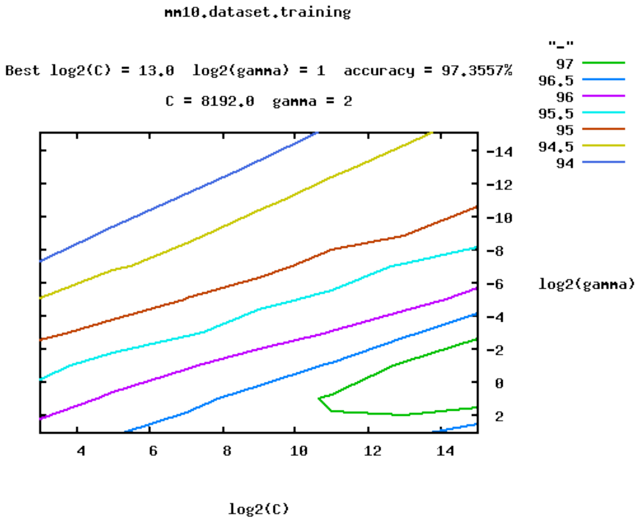

Figure 6: GRCm38 data using longest ORF.

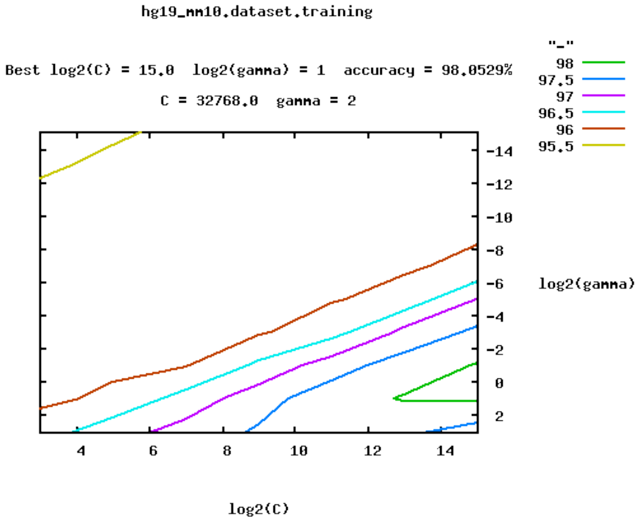

Figure 7: GRCh37 and GRCm38 data using first ORF.

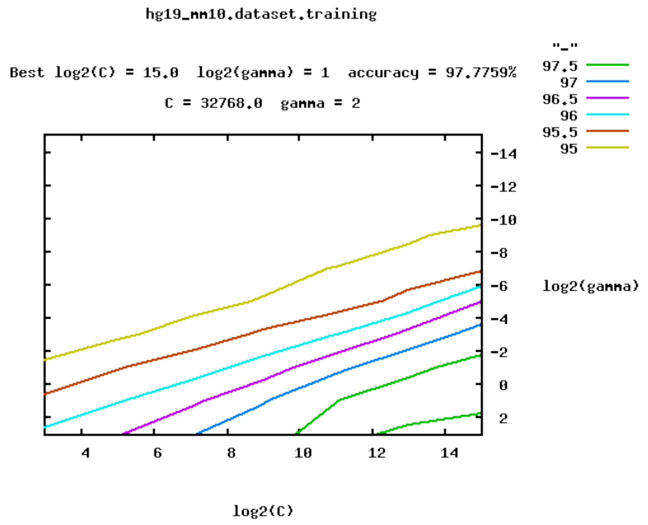

Figure 8: GRCh37 and GRCm38 data using longest ORF.

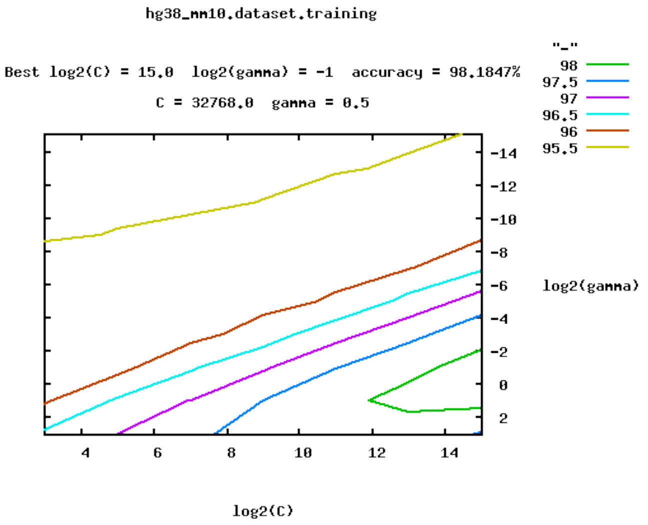

Figure 9: GRCh38 and GRCm38 data using first ORF.

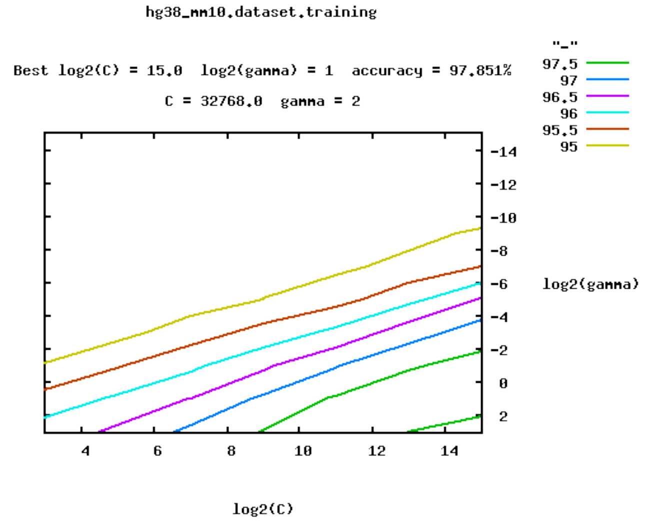

Figure 10: GRCh38 and GRCm38 data using longest ORF.

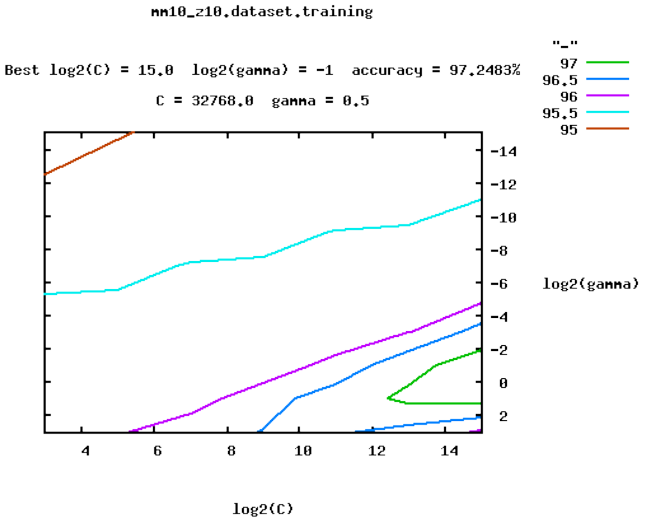

Figure 11: GRCm38 and GRCz10 data using first ORF.
